# Supplementary material for: Research on rock breaking mechanism of PDC cutter under the action of ultrasonic vibration
Source: PLoS One. 2024 Dec 17;19(12):e0308491. doi: 10.1371/journal.pone.0308491 (PMC11651593; doi:10.1371/journal.pone.0308491)
Supplement: S1 File — (DOCX) [file pone.0308491.s001.docx]

Fig 3. Data

Test data

| Strain (‰) | Stess(MPa) |
| --- | --- |
| 0 | 0 |
| 0.7 | 1.03938 |
| 0.84 | 1.24726 |
| 0.98 | 1.45513 |
| 1.12 | 1.66301 |
| 1.26 | 1.87088 |
| 1.4 | 2.1827 |
| 1.68 | 2.39057 |
| 1.82143 | 2.59845 |
| 1.96143 | 2.80632 |
| 2.10143 | 3.0142 |
| 2.24143 | 3.22208 |
| 2.38143 | 3.42995 |
| 2.52143 | 3.63783 |
| 2.66143 | 3.8457 |
| 2.80143 | 4.15752 |
| 3.08143 | 4.67721 |
| 3.22143 | 5.09296 |
| 3.36143 | 5.30083 |
| 3.50143 | 5.50871 |
| 3.64143 | 5.71659 |
| 3.78143 | 6.0284 |
| 3.92143 | 6.34021 |
| 4.06143 | 6.65203 |
| 4.34143 | 7.06778 |
| 4.48143 | 7.27565 |
| 4.62143 | 7.48353 |
| 4.76143 | 7.69141 |
| 4.90143 | 7.89928 |
| 5.04143 | 8.00322 |
| 5.18143 | 8.31503 |
| 5.32143 | 8.83472 |
| 5.46286 | 9.77016 |
| 5.74286 | 11.12136 |
| 5.88286 | 12.26468 |
| 6.02286 | 13.20012 |
| 6.16286 | 14.13556 |
| 6.30286 | 15.071 |
| 6.44286 | 16.11038 |
| 6.58286 | 17.04582 |
| 6.72286 | 18.29307 |
| 6.86286 | 19.95608 |
| 7.14286 | 22.13878 |
| 7.28286 | 23.80178 |
| 7.42286 | 25.46479 |
| 7.56286 | 27.23174 |
| 7.70286 | 28.89474 |
| 7.84286 | 30.76563 |
| 7.98286 | 32.84438 |
| 8.12286 | 35.23496 |
| 8.40286 | 38.24916 |
| 8.54286 | 41.15942 |
| 8.68286 | 43.13424 |
| 8.82286 | 45.10906 |
| 8.96429 | 47.49963 |
| 9.10429 | 50.09808 |
| 9.24429 | 52.59259 |
| 9.38429 | 54.98316 |
| 9.52429 | 58.30917 |
| 9.80429 | 62.67457 |
| 9.94429 | 64.85727 |
| 10.08429 | 67.24783 |
| 10.22429 | 69.84628 |
| 10.36429 | 57.16586 |
| 10.50429 | 61.32338 |
| 10.64429 | 62.98638 |
| 10.78429 | 65.27302 |
| 11.06429 | 67.03996 |
| 11.20429 | 69.53447 |
| 11.34429 | 71.09354 |
| 11.48429 | 71.92504 |
| 11.62429 | 73.1723 |
| 11.76429 | 75.04318 |
| 11.90429 | 76.81013 |
| 12.04429 | 77.53769 |
| 12.32429 | 77.12194 |
| 12.46429 | 78.99282 |
| 12.60571 | 82.2149 |
| 12.74571 | 83.8779 |
| 12.88571 | 86.26848 |
| 13.02571 | 88.13936 |
| 13.16571 | 88.86692 |
| 13.30571 | 90.84175 |
| 13.44571 | 93.12838 |
| 13.72571 | 96.35045 |
| 13.86571 | 99.98828 |
| 14.00571 | 102.067 |
| 14.14571 | 104.2497 |
| 14.28571 | 106.0167 |
| 14.42571 | 98.84496 |
| 14.56571 | 95.83076 |
| 14.70571 | 97.07802 |
| 14.84571 | 99.57253 |
| 15.12571 | 103.834 |
| 15.26572 | 106.7442 |
| 15.40571 | 107.9915 |
| 15.54571 | 109.1348 |
| 15.68571 | 110.486 |
| 15.82571 | 112.0451 |
| 15.96571 | 112.6687 |
| 16.10714 | 112.253 |
| 16.24714 | 112.149 |
| 16.52714 | 113.2923 |
| 16.66714 | 113.6042 |
| 16.80714 | 112.6687 |
| 16.94714 | 110.59 |
| 17.08714 | 109.3427 |
| 17.36714 | 46.25238 |

Simulation data

| Strain (‰) | Stess(MPa) |
| --- | --- |
| 0 | 0 |
| 16.59592 | 112.57524 |
| 16.9481 | 111.61404 |
| 17.30028 | 110.21796 |
| 17.65246 | 105.4602 |
| 18.00464 | 96.86529 |
| 18.35682 | 74.27342 |
| 18.709 | 38.10074 |

Fig 4. Data

| Time | Amplitude |
| --- | --- |
| 1.50E-06 | 7.49525 |
| 3.00E-06 | 14.72498 |
| 4.50E-06 | 21.43307 |
| 6.00E-06 | 27.38189 |
| 7.50E-06 | 32.36068 |
| 9.00E-06 | 36.19308 |
| 1.05E-05 | 38.74333 |
| 1.20E-05 | 39.92107 |
| 1.35E-05 | 39.68459 |
| 1.50E-05 | 38.04226 |
| 1.65E-05 | 35.05227 |
| 1.80E-05 | 30.82053 |
| 1.95E-05 | 25.49696 |
| 2.10E-05 | 19.27015 |
| 2.25E-05 | 12.36068 |
| 2.40E-05 | 5.01333 |
| 2.55E-05 | -2.51162 |
| 2.70E-05 | -9.9476 |
| 2.85E-05 | -17.0312 |
| 3.00E-05 | -23.5114 |
| 3.15E-05 | -29.1587 |
| 3.30E-05 | -33.7731 |
| 3.45E-05 | -37.1911 |
| 3.60E-05 | -39.2915 |
| 3.75E-05 | -40 |
| 3.90E-05 | -39.2915 |
| 4.05E-05 | -37.1911 |
| 4.20E-05 | -33.7731 |
| 4.35E-05 | -29.1587 |
| 4.50E-05 | -23.5114 |
| 4.65E-05 | -17.0312 |
| 4.80E-05 | -9.9476 |
| 4.95E-05 | -2.51162 |
| 5.10E-05 | 5.01333 |
| 5.25E-05 | 12.36068 |
| 5.40E-05 | 19.27015 |
| 5.55E-05 | 25.49696 |
| 5.70E-05 | 30.82053 |
| 5.85E-05 | 35.05227 |
| 6.00E-05 | 38.04226 |
| 6.15E-05 | 39.68459 |
| 6.30E-05 | 39.92107 |
| 6.45E-05 | 38.74333 |
| 6.60E-05 | 36.19308 |
| 6.75E-05 | 32.36068 |
| 6.90E-05 | 27.38189 |
| 7.05E-05 | 21.43307 |
| 7.20E-05 | 14.72498 |
| 7.35E-05 | 7.49525 |
| 7.50E-05 | 1.47E-14 |
| 7.65E-05 | -7.49525 |
| 7.80E-05 | -14.725 |
| 7.95E-05 | -21.4331 |
| 8.10E-05 | -27.3819 |
| 8.25E-05 | -32.3607 |
| 8.40E-05 | -36.1931 |
| 8.55E-05 | -38.7433 |
| 8.70E-05 | -39.9211 |
| 8.85E-05 | -39.6846 |
| 9.00E-05 | -38.0423 |
| 9.15E-05 | -35.0523 |
| 9.30E-05 | -30.8205 |
| 9.45E-05 | -25.497 |
| 9.60E-05 | -19.2702 |
| 9.75E-05 | -12.3607 |
| 9.90E-05 | -5.01333 |
| 1.01E-04 | 2.51162 |
| 1.02E-04 | 9.9476 |
| 1.04E-04 | 17.03117 |
| 1.05E-04 | 23.51141 |
| 1.07E-04 | 29.15874 |
| 1.08E-04 | 33.77312 |
| 1.10E-04 | 37.19106 |
| 1.11E-04 | 39.29149 |
| 1.13E-04 | 40 |
| 1.14E-04 | 39.29149 |
| 1.16E-04 | 37.19106 |
| 1.17E-04 | 33.77312 |
| 1.19E-04 | 29.15874 |
| 1.20E-04 | 23.51141 |
| 1.22E-04 | 17.03117 |
| 1.23E-04 | 9.9476 |
| 1.25E-04 | 2.51162 |
| 1.26E-04 | -5.01333 |
| 1.28E-04 | -12.3607 |
| 1.29E-04 | -19.2702 |
| 1.31E-04 | -25.497 |
| 1.32E-04 | -30.8205 |
| 1.34E-04 | -35.0523 |
| 1.35E-04 | -38.0423 |
| 1.37E-04 | -39.6846 |
| 1.38E-04 | -39.9211 |
| 1.40E-04 | -38.7433 |
| 1.41E-04 | -36.1931 |
| 1.43E-04 | -32.3607 |
| 1.44E-04 | -27.3819 |
| 1.46E-04 | -21.4331 |
| 1.47E-04 | -14.725 |
| 1.49E-04 | -7.49525 |

Fig 5. Data

| Time | 0kHz | 20kHz | 25kHz | 30kHz | 35kHz | 40kHz |
| --- | --- | --- | --- | --- | --- | --- |
| 0 | 0 | 0 | 0 | 0 | 0 | 0 |
| 0.0008005 | 1413.3818 | 2468.1367 | 2148.0347 | 1800.0975 | 1776.2903 | 1814.1851 |
| 0.0016007 | 1330.3733 | 379.74927 | 310.05899 | 419.8515 | 477.32132 | 488.31326 |
| 0.0024008 | 313.81168 | 617.5202 | 629.15485 | 642.22009 | 402.27954 | 688.61627 |
| 0.0032006 | 389.13089 | 483.5069 | 558.85272 | 750.89081 | 287.95593 | 455.67059 |
| 0.004 | 1222.3026 | 400.57767 | 440.78015 | 962.51709 | 403.11337 | 80.856369 |
| 0.0048004 | 1266.873 | 439.81134 | 445.90396 | 632.50775 | 146.19043 | 64.976593 |
| 0.0056008 | 1258.1558 | 485.58533 | 668.13928 | 942.68774 | 439.84729 | 367.93689 |
| 0.0064003 | 884.78906 | 665.99384 | 634.93311 | 811.77722 | 387.26477 | 255.89716 |
| 0.0072007 | 949.54608 | 786.19446 | 822.78467 | 874.97791 | 915.45239 | 309.62836 |
| 0.0080002 | 1235.8081 | 1053.0713 | 923.72522 | 1187.5767 | 1065.795 | 362.93805 |
| 0.0088006 | 1321.8414 | 1116.0852 | 1306.9885 | 1122.47 | 1379.4232 | 1154.3398 |
| 0.0096 | 1265.1984 | 1130.4032 | 892.77692 | 1014.3425 | 1545.0532 | 1045.0153 |
| 0.0104004 | 1274.0828 | 1045.8217 | 786.11475 | 881.93689 | 1543.7834 | 1231.496 |
| 0.0112008 | 1155.0371 | 652.5155 | 774.90411 | 513.93591 | 1094.2241 | 1265.9159 |
| 0.0120003 | 938.73376 | 888.99927 | 622.88721 | 714.66888 | 1001.9932 | 852.55859 |
| 0.0128007 | 992.33191 | 624.41913 | 357.80273 | 439.0351 | 1107.4316 | 177.30135 |
| 0.0136002 | 725.47229 | 844.08521 | 475.80859 | 485.8764 | 808.08008 | 346.10687 |
| 0.0144006 | 1155.3762 | 578.64746 | 404.03479 | 450.10635 | 470.40115 | 330.20691 |
| 0.0152 | 818.11993 | 379.76245 | 298.57678 | 579.80945 | 256.87866 | 250.39929 |
| 0.0160004 | 997.8681 | 754.25214 | 753.22375 | 902.70386 | 610.64117 | 518.83289 |
| 0.0168008 | 1334.3922 | 819.41687 | 1078.614 | 944.74072 | 545.08679 | 346.18478 |
| 0.0176003 | 1107.6595 | 588.01758 | 1111.8994 | 1094.1954 | 843.33887 | 641.78015 |
| 0.0184007 | 748.6875 | 825.52338 | 1344.4131 | 1073.2384 | 826.52747 | 872.11957 |
| 0.0192002 | 1099.2109 | 1216.9117 | 1077.3228 | 1557.2195 | 917.62903 | 1063.2881 |
| 0.0200006 | 1236.8979 | 713.52881 | 1095.2668 | 1226.7583 | 1314.3208 | 1018.1835 |
| 0.0208 | 1014.0304 | 999.31274 | 786.39648 | 810.38184 | 856.46436 | 1059.7549 |
| 0.0216004 | 1335.0088 | 829.47058 | 858.50165 | 523.14691 | 877.59802 | 997.45374 |
| 0.0224008 | 1193.4659 | 755.35126 | 1100.4568 | 1192.9927 | 424.51483 | 833.17865 |
| 0.0232003 | 958.30322 | 775.09094 | 699.31335 | 914.61499 | 316.06595 | 299.00717 |
| 0.0240007 | 763.91058 | 365.2348 | 680.19226 | 738.25366 | 589.07776 | 219.59494 |
| 0.0248002 | 884.22021 | 435.25348 | 447.5657 | 820.53607 | 677.38794 | 271.95288 |
| 0.0256006 | 842.99194 | 793.75024 | 602.29468 | 456.51971 | 138.2413 | 488.91699 |
| 0.0264 | 1263.3134 | 742.7746 | 696.08643 | 677.18341 | 588.73242 | 722.86633 |
| 0.0272004 | 1521.2975 | 913.66833 | 888.79773 | 755.23511 | 693.52002 | 778.62915 |
| 0.0280008 | 1429.9828 | 1305.4883 | 953.49963 | 1353.3915 | 1089.8525 | 892.89246 |
| 0.0288003 | 915.26001 | 1154.4336 | 1162.9159 | 1254.9009 | 1334.5518 | 1134.1852 |
| 0.0296007 | 1138.358 | 1179.396 | 1522.5079 | 1155.7031 | 1104.6019 | 947.84338 |
| 0.0304002 | 1011.6417 | 1297.2458 | 827.47473 | 605.1532 | 1320.4749 | 1362.2617 |
| 0.0312006 | 1288.2681 | 641.19739 | 994.44043 | 1150.7629 | 746.97961 | 1547.5952 |
| 0.032 | 1340.1599 | 674.19775 | 916.67151 | 759.72107 | 1195.9237 | 349.48312 |
| 0.0328004 | 1126.9258 | 1044.2577 | 555.55511 | 642.71973 | 766.99878 | 379.10483 |
| 0.0336008 | 905.7074 | 726.57184 | 468.80804 | 498.44727 | 190.02707 | 269.89771 |
| 0.0344003 | 843.69617 | 631.18048 | 485.72147 | 501.56787 | 442.80188 | 312.87897 |
| 0.0352007 | 1144.4165 | 471.97949 | 457.71356 | 458.76227 | 582.16431 | 412.48706 |
| 0.0360002 | 741.84631 | 837.04614 | 803.94592 | 785.40076 | 630.09045 | 284.94287 |
| 0.0368006 | 1010.7521 | 816.77942 | 987.21594 | 1070.3513 | 899.883 | 458.80515 |
| 0.0376 | 1440.7747 | 735.25085 | 957.83344 | 890.7926 | 987.2674 | 574.75391 |
| 0.0384004 | 1469.2622 | 1291.1877 | 1092.5872 | 1007.7926 | 1059.2854 | 845.9201 |
| 0.0392008 | 1087.7167 | 1004.2596 | 1067.7583 | 811.75391 | 1142.9838 | 841.48267 |
| 0.0400003 | 1057.6323 | 1173.4979 | 1147.9954 | 1120.6962 | 901.46558 | 1246.1887 |
| 0.0408007 | 866.22693 | 1115.6033 | 907.1106 | 876.60095 | 1071.6553 | 1355.0189 |
| 0.0416002 | 802.91235 | 819.6076 | 549.55389 | 1032.9519 | 1114.9143 | 1149.0363 |
| 0.0424006 | 1145.762 | 609.96948 | 420.48444 | 787.73608 | 441.21338 | 422.84845 |
| 0.0432 | 1112.3279 | 599.76233 | 826.94788 | 783.22827 | 475.31577 | 455.66388 |
| 0.0440004 | 856.45288 | 417.01764 | 740.2135 | 461.13889 | 386.37335 | 654.27515 |
| 0.0448008 | 951.59912 | 481.33301 | 490.01953 | 443.85703 | 431.0531 | 242.58893 |
| 0.0456003 | 1204.8805 | 651.02905 | 577.19403 | 728.54663 | 585.69153 | 510.15771 |
| 0.0464007 | 774.4184 | 880.63385 | 707.30273 | 799.69647 | 867.02783 | 378.13376 |
| 0.0472002 | 993.77692 | 1087.4309 | 1149.0082 | 771.91992 | 864.41608 | 572.65857 |
| 0.0480006 | 1335.24 | 1225.7704 | 940.172 | 1346.7163 | 950.46826 | 488.45148 |
| 0.0488 | 993.61487 | 1104.345 | 874.29852 | 1023.0185 | 1311.1193 | 1223.8239 |
| 0.0496004 | 1460.5688 | 1071.0369 | 1158.6969 | 819.75098 | 1249.0811 | 894.15356 |
| 0.0504008 | 1224.4771 | 844.77545 | 1279.3743 | 1017.8263 | 1210.3052 | 603.79919 |
| 0.0512003 | 682.88873 | 915.50854 | 1018.0767 | 1091.071 | 1365.2477 | 1231.8398 |
| 0.0520007 | 693.36096 | 1064.8959 | 713.07483 | 961.97205 | 731.40863 | 659.49829 |
| 0.0528002 | 1271.5818 | 693.29761 | 861.15106 | 904.12787 | 410.44913 | 556.75647 |
| 0.0536006 | 1000.9775 | 497.9046 | 595.3241 | 852.36176 | 484.27789 | 425.31763 |
| 0.0544 | 843.22101 | 482.00598 | 466.24939 | 466.57916 | 532.46216 | 635.46045 |
| 0.0552004 | 1068.1599 | 757.22894 | 597.73041 | 604.15302 | 654.90747 | 301.18292 |
| 0.0560008 | 1408.9625 | 649.23016 | 515.94434 | 735.70227 | 1005.5162 | 302.54956 |
| 0.0568003 | 1130.6169 | 879.49487 | 1017.1621 | 1147.2437 | 1144.9868 | 422.64331 |
| 0.0576007 | 1009.7851 | 1222.303 | 1337.7242 | 1284.2317 | 1162.8413 | 706.94342 |
| 0.0584002 | 1132.2871 | 848.79565 | 1151.2537 | 1010.3223 | 956.11255 | 1080.7961 |
| 0.0592006 | 1284.9655 | 1016.8386 | 987.52734 | 1390.8074 | 1432.262 | 982.27966 |
| 0.06 | 987.026 | 968.61005 | 876.52429 | 1521.9625 | 760.35205 | 1059.0839 |
| 0.0608004 | 925.99292 | 724.82013 | 867.94397 | 964.74207 | 1033.3767 | 1567.0947 |
| 0.0616008 | 1028.0503 | 641.18079 | 760.12012 | 900.05737 | 844.73492 | 411.11627 |
| 0.0624003 | 815.57477 | 680.85474 | 909.01111 | 594.16742 | 501.9845 | 793.73816 |
| 0.0632007 | 765.91382 | 465.39478 | 724.36401 | 490.2569 | 549.44727 | 1184.7227 |
| 0.0640007 | 884.8147 | 396.38953 | 386.25391 | 479.17953 | 533.16406 | 1005.3104 |
| 0.0648007 | 559.55994 | 518.06671 | 489.62747 | 464.0929 | 770.99524 | 512.59845 |
| 0.0656007 | 1473.5107 | 562.87378 | 659.25732 | 716.51672 | 835.25708 | 386.23987 |
| 0.0664007 | 1400.2354 | 812.31921 | 582.11078 | 1008.3125 | 933.23303 | 85.417046 |
| 0.0672008 | 1382.4841 | 1040.0283 | 831.96698 | 1295.4626 | 1151.3899 | 54.438171 |
| 0.0680008 | 1030.5173 | 887.57043 | 1055.9446 | 1096.6995 | 1095.4417 | 383.31506 |
| 0.0688008 | 878.04987 | 1089.2866 | 1115.1721 | 775.96344 | 981.38336 | 855.86255 |
| 0.0696008 | 975.83258 | 1340.172 | 1166.6875 | 941.28394 | 1340.1619 | 1280.7631 |
| 0.0704008 | 761.89917 | 958.35535 | 1136.7614 | 1209.8882 | 237.33791 | 1194.9946 |
| 0.0712008 | 998.33667 | 840.69128 | 992.52283 | 446.10855 | 260.96686 | 1382.5339 |
| 0.0720008 | 1222.0754 | 718.56427 | 677.30054 | 877.60791 | 964.66235 | 557.70892 |
| 0.0728008 | 993.7688 | 711.07739 | 596.62067 | 566.61328 | 343.33234 | 716.90076 |
| 0.0736008 | 1073.0898 | 354.38293 | 790.58118 | 626.41125 | 582.2066 | 410.41748 |
| 0.0744008 | 1049.9901 | 644.33801 | 615.13232 | 466.25476 | 289.33435 | 224.04437 |
| 0.0752008 | 955.11719 | 367.4834 | 396.54132 | 429.76797 | 752.26929 | 170.25513 |
| 0.0760008 | 677.50488 | 434.57141 | 548.31555 | 576.98584 | 1162.2236 | 384.05658 |
| 0.0768008 | 1301.2062 | 745.2077 | 771.97083 | 842.67334 | 1067.4768 | 1007.3683 |
| 0.0776008 | 1265.7377 | 1172.1665 | 524.66541 | 818.05566 | 1026.0793 | 847.43054 |
| 0.0784008 | 1388.1748 | 977.88708 | 1109.1257 | 957.58466 | 870.77686 | 767.37305 |
| 0.0792008 | 1142.936 | 1097.7197 | 1003.0607 | 1059.1733 | 1358.595 | 628.13257 |
| 0.08 | 1228.1826 | 1125.4176 | 1039.5583 | 714.86877 | 1136.2666 | 1404.4043 |

Fig 7. Data

|  | 0kHz | 20kHz | 25kHz | 30kHz | 35kHz | 40kHz |
| --- | --- | --- | --- | --- | --- | --- |
| **Average Force** | 1055.2344 | 809.54121 | 808.2573 | 843.21955 | 815.29538 | 683.82202 |
| **MSE** | 31.18616 | 24.25009 | 26.34729 | 28.97419 | 29.8616 | 28.78417 |

Fig 9. Data

| Time | 0kHZ | 20kHz | 25kHz | 30kHz | 35kHz | 40kHz |
| --- | --- | --- | --- | --- | --- | --- |
| 0 | 27 | 27 | 27 | 27 | 27 | 27 |
| 8.00E-04 | 27.04179 | 28.14541 | 28.50114 | 28.70511 | 29.00457 | 29.06462 |
| 0.0016 | 27.53249 | 32.24503 | 33.09028 | 34.56414 | 35.39354 | 35.89307 |
| 0.0024 | 27.7673 | 32.13231 | 35.3638 | 37.12768 | 38.27606 | 41.52253 |
| 0.0032 | 28.2543 | 36.36581 | 39.63024 | 43.15516 | 45.60862 | 48.98125 |
| 0.004 | 28.54226 | 38.52779 | 42.36486 | 47.2525 | 50.09816 | 52.93855 |
| 0.0048 | 28.78075 | 41.1032 | 45.41661 | 50.54015 | 52.79398 | 56.95065 |
| 0.0056 | 28.89429 | 43.46264 | 47.4748 | 53.88713 | 56.06518 | 59.48857 |
| 0.0064 | 29.09062 | 45.31708 | 49.07433 | 55.79947 | 58.99042 | 62.00881 |
| 0.0072 | 29.37515 | 46.66496 | 50.57698 | 57.67091 | 60.64814 | 63.65553 |
| 0.008 | 29.63745 | 47.69814 | 52.41993 | 59.27552 | 63.46716 | 67.98912 |
| 0.0088 | 29.70431 | 48.96276 | 53.59814 | 61.55885 | 65.86649 | 67.89781 |
| 0.0096 | 29.89154 | 50.20795 | 55.3217 | 63.86313 | 66.07028 | 69.65112 |
| 0.0104 | 30.0619 | 50.88816 | 56.34464 | 65.45868 | 69.4941 | 70.93095 |
| 0.0112 | 30.248 | 51.88658 | 57.73089 | 68.0661 | 71.94963 | 72.52425 |
| 0.012 | 30.42169 | 53.44931 | 58.89581 | 69.21001 | 74.63882 | 75.86455 |
| 0.0128 | 30.6316 | 54.69096 | 60.15021 | 70.70654 | 76.41748 | 77.85963 |
| 0.0136 | 30.60155 | 55.57907 | 61.31467 | 72.31427 | 77.63896 | 79.78357 |
| 0.0144 | 30.69382 | 56.48885 | 61.87167 | 73.09166 | 79.3998 | 82.25027 |
| 0.0152 | 30.89375 | 56.256 | 62.62938 | 74.58675 | 80.02286 | 83.1297 |
| 0.016 | 31.0069 | 56.85558 | 63.64291 | 76.06422 | 82.04265 | 84.48628 |
| 0.0168 | 31.04129 | 57.88093 | 64.7943 | 76.90135 | 83.95498 | 85.39828 |
| 0.0176 | 31.20958 | 59.03699 | 65.4469 | 77.18936 | 83.68307 | 86.7101 |
| 0.0184 | 31.3697 | 60.51444 | 65.82837 | 78.12422 | 84.97047 | 86.53976 |
| 0.0192 | 31.46642 | 60.28563 | 66.66943 | 79.75948 | 86.4893 | 87.61168 |
| 0.02 | 31.5204 | 61.08131 | 67.75128 | 81.44144 | 87.88362 | 88.87068 |
| 0.0208 | 31.59974 | 62.08929 | 68.77982 | 82.91029 | 89.18599 | 90.46729 |
| 0.0216 | 31.66462 | 62.42681 | 70.20322 | 83.1356 | 90.31845 | 91.68031 |
| 0.0224 | 31.85138 | 62.67807 | 70.89653 | 84.10983 | 91.94635 | 93.79092 |
| 0.0232 | 32.03396 | 63.40002 | 71.66163 | 84.77747 | 93.73333 | 95.32684 |
| 0.024 | 32.0325 | 64.42772 | 72.00082 | 86.39333 | 94.31116 | 94.6865 |
| 0.0248 | 31.96061 | 65.27119 | 72.8036 | 87.50041 | 94.4174 | 97.54373 |
| 0.0256 | 32.10337 | 66.0136 | 73.90775 | 88.00661 | 93.7935 | 97.95202 |
| 0.0264 | 32.21757 | 66.64756 | 73.94366 | 88.6897 | 96.13868 | 98.79167 |
| 0.0272 | 32.2641 | 67.04332 | 74.06592 | 89.10555 | 95.89465 | 99.58443 |
| 0.028 | 32.23195 | 67.11041 | 74.46349 | 89.86281 | 97.86716 | 100.0044 |
| 0.0288 | 32.41106 | 67.00597 | 74.7291 | 90.67168 | 99.19968 | 100.1812 |
| 0.0296 | 32.57619 | 67.4299 | 75.70097 | 91.20967 | 100.1687 | 101.3649 |
| 0.0304 | 32.62751 | 68.1465 | 77.04288 | 92.41002 | 101.5197 | 102.8865 |
| 0.0312 | 32.60239 | 68.57004 | 77.49471 | 92.59311 | 102.4707 | 103.9113 |
| 0.032 | 32.67719 | 69.39537 | 78.0954 | 94.52837 | 102.891 | 105.0728 |
| 0.0328 | 32.86537 | 69.82897 | 78.77968 | 95.92173 | 103.0135 | 105.5691 |
| 0.0336 | 32.97044 | 70.16823 | 79.60741 | 96.50706 | 102.3497 | 106.9463 |
| 0.0344 | 32.94998 | 70.5612 | 79.90573 | 97.59908 | 105.0929 | 108.2516 |
| 0.0352 | 32.90794 | 71.42107 | 80.75804 | 97.3094 | 106.4747 | 106.7361 |
| 0.036 | 32.95993 | 71.80707 | 82.01256 | 98.83443 | 107.3971 | 107.4211 |
| 0.0368 | 33.05861 | 71.79124 | 82.98897 | 99.80295 | 107.0266 | 108.6281 |
| 0.0376 | 33.16781 | 72.45374 | 83.48405 | 99.96187 | 107.9992 | 110.6766 |
| 0.0384 | 33.12619 | 73.17356 | 83.35075 | 100.2233 | 109.28 | 111.7146 |
| 0.0392 | 33.16433 | 73.58055 | 83.50485 | 100.855 | 110.0883 | 111.9936 |
| 0.04 | 33.35883 | 74.10275 | 84.17963 | 101.4099 | 111.5164 | 111.7362 |
| 0.0408 | 33.44184 | 74.37667 | 85.22603 | 102.6237 | 111.3073 | 112.7073 |
| 0.0416 | 33.4723 | 74.71376 | 86.23194 | 103.5566 | 111.9968 | 113.8764 |
| 0.0424 | 33.48794 | 75.16983 | 86.93172 | 104.1389 | 113.6526 | 116.7349 |
| 0.0432 | 33.61756 | 75.53992 | 87.56102 | 105.2632 | 114.6426 | 117.04 |
| 0.044 | 33.77702 | 75.90683 | 87.52737 | 106.2342 | 115.0502 | 117.2255 |
| 0.0448 | 33.74751 | 75.8829 | 87.54434 | 106.6026 | 115.6917 | 117.9955 |
| 0.0456 | 33.68575 | 76.05948 | 88.33597 | 107.3803 | 116.0046 | 118.2264 |
| 0.0464 | 33.83355 | 75.96185 | 88.75956 | 107.3216 | 116.283 | 119.6756 |
| 0.0472 | 33.85968 | 76.29407 | 89.36452 | 107.6902 | 117.0504 | 121.259 |
| 0.048 | 33.93677 | 76.92188 | 89.41747 | 107.4702 | 117.349 | 122.0687 |
| 0.0488 | 33.96445 | 77.14469 | 89.89931 | 108.2276 | 117.9556 | 122.4466 |
| 0.0496 | 33.79703 | 77.21561 | 90.71417 | 109.6287 | 119.3731 | 122.9249 |
| 0.0504 | 33.95953 | 77.55288 | 91.38634 | 110.3934 | 119.9023 | 123.1113 |
| 0.0512 | 34.12273 | 77.72235 | 91.51295 | 110.4327 | 119.8037 | 122.8639 |
| 0.052 | 34.16365 | 78.68141 | 91.83323 | 111.7079 | 121.8599 | 125.5939 |
| 0.0528 | 33.98542 | 79.24342 | 92.11957 | 112.3643 | 122.5206 | 126.1438 |
| 0.0536 | 34.22936 | 79.80888 | 92.85869 | 112.8055 | 121.8002 | 126.0778 |
| 0.0544 | 34.36058 | 80.73453 | 93.0797 | 113.4313 | 122.8469 | 126.0749 |
| 0.0552 | 34.3465 | 81.29936 | 93.20805 | 114.2704 | 124.2139 | 126.7276 |
| 0.056 | 34.34361 | 81.96779 | 93.35858 | 114.5738 | 122.6336 | 127.3832 |
| 0.0568 | 34.39395 | 82.38316 | 93.33611 | 114.5823 | 123.2399 | 129.1409 |
| 0.0576 | 34.50091 | 82.46526 | 93.44185 | 115.1263 | 124.1612 | 129.3295 |
| 0.0584 | 34.59177 | 82.11427 | 94.09073 | 116.2846 | 124.3327 | 128.6922 |
| 0.0592 | 34.65529 | 82.27194 | 94.79341 | 116.3473 | 125.3409 | 129.5043 |
| 0.06 | 34.60559 | 82.40899 | 95.71138 | 117.3632 | 127.1601 | 130.1969 |
| 0.0608 | 34.56781 | 83.31568 | 96.28968 | 117.978 | 128.329 | 129.2884 |
| 0.0616 | 34.73416 | 83.32027 | 96.37753 | 118.6668 | 129.0685 | 132.2197 |
| 0.0624 | 34.92936 | 84.12631 | 97.0489 | 119.216 | 130.3339 | 132.5986 |
| 0.0632 | 34.83308 | 84.15799 | 97.02399 | 120.2096 | 130.1946 | 133.1111 |
| 0.064 | 34.81465 | 84.4978 | 97.412 | 120.8074 | 130.6005 | 134.1994 |
| 0.0648 | 35.00446 | 84.62263 | 98.19551 | 120.7698 | 130.8232 | 135.5278 |
| 0.0656 | 34.99013 | 84.7896 | 98.89732 | 121.504 | 132.448 | 136.2312 |
| 0.0664 | 34.93739 | 85.20374 | 99.25462 | 122.1 | 131.2928 | 136.4217 |
| 0.0672 | 34.93923 | 85.79076 | 99.11264 | 121.7257 | 131.1129 | 135.788 |
| 0.068 | 35.13087 | 86.30057 | 99.3604 | 121.9331 | 131.7895 | 136.1127 |
| 0.0688 | 35.25963 | 86.79729 | 100.041 | 123.0353 | 132.3193 | 135.7385 |
| 0.0696 | 35.36467 | 86.80383 | 100.6861 | 123.6978 | 134.1028 | 135.887 |
| 0.0704 | 35.40888 | 86.76318 | 101.7764 | 124.2699 | 133.6437 | 136.8109 |
| 0.0712 | 35.23565 | 86.87216 | 102.3764 | 124.9401 | 134.684 | 137.4542 |
| 0.072 | 35.2931 | 87.53018 | 102.6633 | 125.2697 | 135.1468 | 139.2984 |
| 0.0728 | 35.45041 | 88.06402 | 103.1002 | 126.6956 | 134.824 | 139.7077 |
| 0.0736 | 35.49599 | 88.74921 | 103.0999 | 127.5705 | 135.9583 | 139.8428 |
| 0.0744 | 35.48195 | 89.57547 | 103.8528 | 127.2782 | 136.005 | 138.6825 |
| 0.0752 | 35.48176 | 89.33405 | 104.24 | 127.8442 | 136.6309 | 140.4573 |
| 0.076 | 35.60825 | 89.19164 | 104.3219 | 128.487 | 136.5626 | 141.8118 |
| 0.0768 | 35.64434 | 89.23582 | 104.3353 | 129.0014 | 137.1788 | 140.9858 |
| 0.0776 | 35.61053 | 88.75493 | 105.0559 | 129.0725 | 138.0302 | 141.0576 |
| 0.0784 | 35.49899 | 88.46635 | 105.6998 | 129.0015 | 138.58 | 141.7728 |
| 0.0792 | 35.60706 | 88.83656 | 105.2073 | 130.0453 | 138.6654 | 142.0233 |
| 0.08 | 35.77669 | 88.73869 | 105.411 | 130.6834 | 140.396 | 141.976 |
