# Supplementary material for: Research on rock breaking mechanism of PDC cutter under the action of ultrasonic vibration
Source: PLoS One. 2024 Dec 17;19(12):e0308491. doi: 10.1371/journal.pone.0308491 (PMC11651593; doi:10.1371/journal.pone.0308491)
Supplement: Attachment — Submitted filename: Response to Editor and Reviewer.docx [file pone.0308491.s002.docx]

**This email was sent to me by the Academic Editor before:**

==============================

PONE-D-24-07628

Research on rock breaking mechanism of PDC cutter under the action of ultrasonic vibration

PLOS ONE

Dear Dr. Zhang,

Thank you for submitting your manuscript to PLOS ONE. After careful consideration, we feel that it has merit but does not fully meet PLOS ONE’s publication criteria as it currently stands. Therefore, we invite you to submit a revised version of the manuscript that addresses the points raised during the review process.

==============================

Dear Authors,

I appreciate you considering PLOS ONE journal for your research publication. After thoroughly reviewing the article, I found it very interesting, it seems to be one of the important numerical simulations performed in Abaqus considering rock breaking mechanics. However, I would appreciate it if you could improve its English. I understand that you are not native English speaker, but at the present form, the article English quality is well below the required standard. There are multiple grammar issues. Most of the text follows a compound-complex sentence structure, with syntax errors, punctuation errors, and missing definite articles. At some places I found subject-verb disagreement. Considering these issues, I recommend contacting some English proofreading company and resubmitting this article with improved and error-free English.

==============================

Dear Editors and Reviewers:

Thank you for your letter and for the Reviewers’ comments concerning our manuscript entitled “Research on rock breaking mechanism of PDC cutter under the action of ultrasonic vibration” (Manuscript Number: PONE-D-24-07628). Those comments are all valuable and very helpful for revising and improving our paper, as well as the important guiding significance to our researches. We have studied comments carefully and have made some correction which we hope to meet with approval. Revised portions are marked in red in the paper. The main correction in the paper and the respond to the Reviewer’s comments are as flowing:

**Responds to** **the reviewer ’s comments:**

**Reviewer #1:**

Thank you for reviewing our manuscript so carefully and thanks very much for your positive evaluation which make us confident in our research. At the same time, your precious suggestions will help us to further improve our research. And according to your comment and suggestions, we have revised our content one by one.

**Comment 1:** This paper does not fully meet PLOS ONE’s publication criteria as it currently stands.

**Response:** Thank you for your valuable and thoughtful comments, we really agree with your viewpoints. According to your helpful advice, we have made modifications to the format of the paper to meet PLOS ONE’s publication criteria. Thank you again.

**Comment 2:** English needs to be greatly improved.

**Response:** Thank you for your valuable advice. We have carefully revised the manuscript according to your comments, and also have re-scrutinized to improve the English by a language polishing service.

Special thanks to you for your good comments and suggestions.

We tried our best to improve the manuscript and made some changes in the manuscript. These changes will not influence the content and framework of the paper. And here we did not list the changes.

We appreciate for the Editors and Reviewers’ warm work earnestly and hope that the correction will meet with approval. We look forward to hearing from you regarding our submission, and we would be glad to respond to any further questions and comments that you may have.

Once again, thank you very much for your comments and suggestions.

Yours

Sincerely

2024.04.26
