# Supplementary material for: Research on rock breaking mechanism of PDC cutter under the action of ultrasonic vibration
Source: PLoS One. 2024 Dec 17;19(12):e0308491. doi: 10.1371/journal.pone.0308491 (PMC11651593; doi:10.1371/journal.pone.0308491)
Supplement: Attachment — Submitted filename: Comments of Reviewer for Manuscript PONE-D-24-07628.docx [file pone.0308491.s003.docx]

**Comments of Reviewer for Manuscript: PONE-D-24-07628**

Dear Authors,

Thank you for submitting your manuscript to PLOS ONE. After careful consideration/ studies, there is significant need to improve the manuscript asper my point of view. So, there is need to submit a revised version of the manuscript that addresses the comments/ points raised during the review process.

Following are the comments / points which are required to be addressed carefully one by one.

**Comment 1**: Line 13-15; the word “great” has used in abstract. Use some other suitable word as engineering/ technical point of view.

**Comment 2**: Line 14; there is mention in abstract as “significantly reduce the cost”. How it is happened and needs clarification as you have not mention any data to support in this context?

**Comment 3**: Use mechanical specific energy (MSE) in Line 22 instead of Line 23 of abstract.

**Comment 4**: Line 30; Use “ultrasonic frequency or ultrasonic vibration frequency” instead of “ultrasonic”.

**Comment 5**: Line 32-34; “and” has used 4 times in one sentence. Improve this sentence and break into small meaningful sentences.

**Comment 6**: Line 72; rotary ultrasonic technology in drilling seems not have relation with rock-breaking with PDC cutter. Please elaborate or improve the words in manuscript.

**Comment 7**: Line 84-88; it is required to elaborate in small sentences for making it more understandable / clarity.

**Comment 8**: Line 108 and Line 120; there is need to add reference as previous research equations have applied.

**Comment 9**: Line 175; the caption of Fig 3 is bold. It is required to improve

**Comment 10**: Line 193; “In this simulation, we only need to investigate”. There is need to improve words.

**Comment 11:** Line 287; there is need give reference of Equation quoted from literature.

**Comment 12:** Fig 3; text is too small and not readable. It is required to improve Fig.3. Also, improve the text of all the figures as per Journal formate.

**Comment 13:** In rock breaking, vibration technology is using since long time. What is the novelty of this research work?

**Comment 14:** What is accuracy of the simulated model as compared to actual experimental results?

**Comment 15:** Before this research, is there any research work is available in literature which compare conventional and ultrasonic rock breaking?

**Comment 16:** In results, there is need to compare similar research works which already reported in literature.

**Comment 17:** How this research work has saved the cost? However, there is not presented any data related to the cost?

**Comment 18:** What is the optimal ultrasonic vibration frequency by considering cutting force, breaking efficiency, temperature? On which frequency value the process is required to be carried out?

**Comment 19:** In conclusions section the last paragraph (2-3 lines) is required to describe practical application of this research work.

**General**

**Comment 20:** The word “the” has used much where also not needed. Please check and remove where not required.

**Comment 21:** Long sentences are not understandable. There is need to make sentences as per English language and grammar.

**Comment 22:** In manuscript, the abbreviations are required to write as per format of journal.

**Comment 23:** English language is required to be improved of complete manuscript.

**Comment 24:** Formatting of the manuscript is required asper journal requirements.

**Comment 25:** References are required to be format as per journal requirements.
