# Supplementary material for: Research on rock breaking mechanism of PDC cutter under the action of ultrasonic vibration
Source: PLoS One. 2024 Dec 17;19(12):e0308491. doi: 10.1371/journal.pone.0308491 (PMC11651593; doi:10.1371/journal.pone.0308491)
Supplement: Attachment — Submitted filename: Response to Editor and Reviewer.docx [file pone.0308491.s004.docx]

Dear Editors and Reviewers:

Thank you for your letter and for the Reviewers’ comments concerning our manuscript entitled “Research on rock breaking mechanism of PDC cutter under the action of ultrasonic vibration” (Manuscript Number: PONE-D-24-07628). Those comments are all valuable and very helpful for revising and improving our paper, as well as the important guiding significance to our researches. We have studied comments carefully and have made some correction which we hope to meet with approval. Revised portions are marked in red in the paper. The main correction in the paper and the respond to the Reviewer’s comments are as flowing:

**Responds to** **the reviewer ’s comments:**

Thank you for reviewing our manuscript so carefully and thanks very much for your positive evaluation which make us confident in our research. At the same time, your precious suggestions will help us to further improve our research. And according to your comment and suggestions, we have revised our content one by one.

**Comment 1**: Line 13-15; the word “great” has used in abstract. Use some other suitable word as engineering/ technical point of view.

**Response:** Thank you for your valuable and thoughtful comments, we really agree with your viewpoints. We have corrected the sentence according to your comment in the revised manuscript. Thank you again.

**Modified sentence**

This is important for practical engineering applications.

**Comment 2**: Line 14; there is mention in abstract as “significantly reduce the cost”. How it is happened and needs clarification as you have not mention any data to support in this context?

**Response:** Thank you for your valuable comments. During the breaking process, when the ultrasonic vibration reaches the optimal frequency, the required cutting force is minimized and the energy consumption is reduced, which significantly reduces the cost of rock breaking and extends the service life of the PDC cutter.

**Comment 3**: Use mechanical specific energy (MSE) in Line 22 instead of Line 23 of abstract.

**Response:** We are sorry for this mistake. We have corrected the sentence according to your comment.

**Modified sentence**

As ultrasonic vibration frequency (20~40kHz) increases, the mechanical specific energy (MSE) initially decreases and then increases. At a frequency of 20~25kHz, the MSE reaches its minimum value.

**Comment 4**: Line 30; Use “ultrasonic frequency or ultrasonic vibration frequency” instead of “ultrasonic”.

**Response:** Thank you for pointing out the problem. According to your comment, we have corrected the “ultrasonic” into “ultrasonic frequency” in the revised manuscript.

**Modified sentence**

**Keywords:** PDC cutter; ultrasonic frequency; temperature; rock breaking efficiency

**Comment 5**: Line 32-34; “and” has used 4 times in one sentence. Improve this sentence and break into small meaningful sentences.

**Response:** Thank you for your valuable advice. We have carefully revised the manuscript according to your comments, and also have re-scrutinized to improve the English by a language polishing service.

**Modified sentence**

With increasing human demand for conventional oil and gas resources, shallow resources are nearing exhaustion. Consequently, the exploration and development are gradually shifting from shallow to deep and ultra-deep wells.

**Comment 6**: Line 72; rotary ultrasonic technology in drilling seems not have relation with rock-breaking with PDC cutter. Please elaborate or improve the words in manuscript.

**Response:** Thank you for your valuable comments. Polycrystalline diamond composite (PDC) bits are the main rock-breaking tools for exploration nowadays, which are widely used in the exploration and development process because of their high rock-breaking efficiency and fast drilling speed. However, the breaking effect of PDC bits is not good in deep hard rock drilling. Therefore, this paper combines ultrasonic vibratory rock-breaking technology with PDC bits to adapt to the challenges posed by deeper formations, so that it can realize cutting and crushing while high-frequency fatigue rock-breaking occurs in deep hard-rock formations, which is of significance for improving the breaking efficiency of hard rock, saving drilling costs and solving the difficult problem of hard-rock drilling.

**Comment 7**: Line 84-88; it is required to elaborate in small sentences for making it more understandable / clarity.

**Response:** Thank you for pointing out the problem, we have made changes in the manuscript.

**Modified sentence**

To date, research on ultrasonic rock breaking has mainly focused on the generation and variation of breaking cracks. However, research on the movement form of the ultrasonic vibration-assisted rock breaking process of the PDC cutter and the dynamic rock breaking efficiency remains relatively scarce, while no research has reported the ultrasonic rock breaking cutting mechanism or the variation trend of the cutting force.

**Comment 8**: Line 108 and Line 120; there is need to add reference as previous research equations have applied.

**Response:** We sincerely appreciate the valuable comments. As suggested by your, we have checked the literature carefully and added references in the revised manuscript.

This study employs the Drucker-Prager (D-P) yield criterion, which can be expressed as follows [19]:

19. Huang J, Zeng B, He Y, Wang X, Qian L, Xia C, et al. Numerical study of rock-breaking mechanism in hard rock with full PDC bit model in compound impact drilling. Energy Reports. 2023;9:3896-909. doi: 10.1016/j.egyr.2023.02.084.

Therefore, the equivalent plastic strain serves as the rock-breaking criterion, that is [20]:

20. Liu W, Zhu X, Li B. The rock breaking mechanism analysis of rotary percussive cutting by single PDC cutter. Arabian Journal of Geosciences. 2018;11(9). doi: 10.1007/s12517-018-3530-6.

**Comment 9**: Line 175; the caption of Fig 3 is bold. It is required to improve

**Response:** Thank you for pointing out the problem. Due to our negligence, the font of the caption was confused. We have carried out all checks and modifications in the manuscript.

**Comment 10**: Line 193; “In this simulation, we only need to investigate”. There is need to improve words.

**Response:** Thank you for your valuable comments. We have re-written this part according to your suggestion.

**Modified sentence**

Since the intrinsic frequency of rocks is 20–40 kHz, we investigated the rock-breaking behavior of PDC cutter at ultrasonic vibration frequencies of 20 kHz, 25 kHz, 30 kHz, 35 kHz, and 40 kHz, respectively.

**Comment 11:** Line 287; there is need give reference of Equation quoted from literature.

**Response:** Thank you for your valuable comments. According to your suggestion, reference has been added.

The expression is as follows[29]:

29. Liu S, Zhou F, Li H, Chen Y, Wang F, Guo C. Experimental Investigation of Hard Rock Breaking Using a Conical Pick Assisted by Abrasive Water Jet. Rock Mechanics and Rock Engineering. 2020;53(9):4221-30. doi: 10.1007/s00603-020-02168-2.

**Comment 12:** Fig 3; text is too small and not readable. It is required to improve Fig.3. Also, improve the text of all the figures as per Journal formate.

**Response:** Thanks for your valuable comments, we have checked the formatting of all the figures in the manuscript and made changes in the new manuscript.

Fig 3. Finite element modeling of PDC cutter with rock

**Comment 13:** In rock breaking, vibration technology is using since long time. What is the novelty of this research work?

**Response:** Thank you for your valuable comments. Currently, most of the research on vibration technology is mainly focused on the theory and macro-experimentation. There has been no relevant discussion among researchers on the cutting mechanism of ultrasonic rock fragmentation and the variation law of cutting force. The innovation point of this paper is to combine the ultrasonic vibration technology with the widely used PDC bits for drilling, and study the changing law of cutting force and MSE of the cutter in the process of ultrasonic vibration rock breaking, in order to improve rock breaking efficiency.

**Comment 14:** What is accuracy of the simulated model as compared to actual experimental results?

**Response:** Thank you for your valuable comments. ABAQUS was used to simulate the uniaxial compression of the granite model. The parameters of the model were calibrated through uniaxial compression experiments. The physical and mechanical parameters used in the model are listed in Table 1. The finite element analysis used a Ф50 mm × 100 mm rock sample and two rigid disks. A completely fixed constraint was applied to the bottom plate, and a compressive displacement load of 5 mm was applied to the upper plate, which only had a z-directional degree-of-freedom. Fig. 7 shows the failure pattern of the rock under uniaxial compression. Fig. 8 shows the stress–strain curves obtained from the numerical simulations and laboratory experiments. These results show that the simulated stress–strain curve is consistent with the results of laboratory tests. The peak strengths of the two curves and the slope of the curve at the elastic stage are basically consistent, and both curves showed the brittle failure characteristics after the peak. A comparison of the numerical simulation and experimental results of uniaxial compression demonstrate that this model can adequately simulate the fracturing and stress–strain behavior of the rock.

**Comment 15:** Before this research, is there any research work is available in literature which compare conventional and ultrasonic rock breaking?

**Response:** Thank you for your valuable comments. Wiercigroch and Fernando et al. have conducted similar experimental studies.

Wiercigroch et al. conducted experimental tests on the efficiency of conventional rock drilling methods and ultrasonic impact. The test results showed that the drilling speed was significantly increased after applying ultrasonic vibratory loads compared to the conventional rotary drilling method. Fernando et al. used a rotary ultrasonic machining unit (RUM) for rock crushing tests, and the mechanical drilling speed was about three times that of percussive drilling.

Wiercigroch M., J. Wojewoda, and A.M. Krivtsov. Dynamics of ultrasonic percussive drilling of hard rocks. Journal of Sound and Vibration, 2005. 280(3 5): 739 757.

Fernando P. Zhang M., and Pei Z.J. Rotary ultrasonic machining of rocks: An experimental investigation. Advances in Mechanical Engineering, 2018. 10(3).

Fernando P., Pei Z.J., and Zhang M. Mechanistic cutting force model for rotary ultrasonic machining of rocks. The International Journal of Advanced Manufacturing Technology, 2020. 109(12): 109 128.

**Comment 16:** In results, there is need to compare similar research works which already reported in literature.

**Response:** Thank you for your valuable comments. Necessary change in the statements has been made in the revised manuscript.

**Comment 17:** How this research work has saved the cost? However, there is not presented any data related to the cost?

**Response:** Thank you for your valuable comments. The cost savings are reflected by the cutting force of the PDC cutter and mechanical specific energy (MSE).

(1) The average cutting force of PDC cutter is 1055.23 N. After applying different frequencies of ultrasonic vibration, the average cutting force of PDC cutter is 809.54 N, 808.26 N, 843.22 N, 815.30 N, and 683.82 N, respectively. These represent decreases of 23.28%, 23.40%, 20.09%, 22.74%, and 35.20%, respectively, compared with conventional rock breaking. Obviously, under ultrasonic vibration rock breaking, the average cutting force decreases significantly, which is conducive to reducing the wear of the drill bits and prolonging its service life. This is the embodiment of material savings.

(2) The MSE of rock breaking under ultrasonic vibration rock breaking frequency of 20kHz, 25kHz, 30kHz, 35kHz, 40kHz is decreased by 22.24%, 15.52%, 7.09%, 4.24%, 7.70%, respectively, compared with that of conventional rock breaking. This is a reflection of reduced energy consumption.

**Comment 18:** What is the optimal ultrasonic vibration frequency by considering cutting force, breaking efficiency, temperature? On which frequency value the process is required to be carried out?

**Response:** Thank you for your valuable comments. The temperature of the PDC cutter increases by 25.27 °C as the vibration frequency increases from 25 kHz to 30 kHz. In addition, the MSE reaches its minimum value between 20 kHz and 25 kHz. Therefore, the results indicate that the optimal frequency for rock breaking is 20–25 kHz under the condition of ensuring a small increase in rock strength.

**Comment 19:** In conclusions section the last paragraph (2-3 lines) is required to describe practical application of this research work.

**Response:** Thank you for your valuable comments. Also thank you for pointing out the problem, which is of great help to the integrity of our paper, we have made changes in the manuscript.

The rock breaking mechanism of PDC cutter under the ultrasonic vibration obtained provides theoretical guidance and technical support for ultrasonic vibration technology to solve hard rock drilling problems in engineering practice, and enables PDC bits to realize a major breakthrough in breaking hard rock.

**General**

**Comment 20:** The word “the” has used much where also not needed. Please check and remove where not required.

**Response:** Thank you for your valuable comments. Necessary change in the statements has been made in the revised manuscript.

**Comment 21:** Long sentences are not understandable. There is need to make sentences as per English language and grammar.

**Response:** Thank you for your valuable comments, we have checked all the grammar

in the manuscript, and also have improved the English writing by Elsevier's language editing service in the revised manuscript.

**Comment 22:** In manuscript, the abbreviations are required to write as per format of journal.

**Response:** Thank you for your comments on our paper. We have corrected it in the revised manuscript.

For example, polycrystalline diamond compact (PDC), mechanical specific energy (MSE)

**Comment 23:** English language is required to be improved of complete manuscript.

**Response:** Thank you for your valuable advice. We have carefully revised the manuscript according to your comments, and also have improved the English writing by Elsevier's language editing service in the revised manuscript.

**Comment 24:** Formatting of the manuscript is required as per journal requirements.

**Response:** Thank you for your valuable comments, we have revised the formatting in the new manuscript according to the journal requirements.

**Comment 25:** References are required to be format as per journal requirements.

**Response:** Thank you for your valuable comments, we have revised the references in the new manuscript according to the journal requirements.

Special thanks to you for your good comments and suggestions.

We tried our best to improve the manuscript and made some changes in the manuscript. These changes will not influence the content and framework of the paper. And here we did not list the changes.

We appreciate for the Editors and Reviewers’ warm work earnestly and hope that the correction will meet with approval. We look forward to hearing from you regarding our submission, and we would be glad to respond to any further questions and comments that you may have.

Once again, thank you very much for your comments and suggestions.

Yours

Sincerely

2024.07.24
